# Mendelian nightmares: the germline-restricted chromosome of songbirds

**DOI:** 10.1007/s10577-022-09688-3

**Published:** 2022-04-13

**Authors:** Pavel Borodin, Augustin Chen, Wolfgang Forstmeier, Simone Fouché, Lyubov Malinovskaya, Yifan Pei, Radka Reifová, Francisco J. Ruiz-Ruano, Stephen A. Schlebusch, Manuelita Sotelo-Muñoz, Anna Torgasheva, Niki Vontzou, Alexander Suh

**Affiliations:** 1 Siberian Department, Russian Academy of Sciences, Institute of Cytology and Genetics, Prospekt Akademika Lavrent’yeva 10, 630090 Novosibirsk, Russia; 2 Department of Organismal Biology – Systematic Biology, Evolutionary Biology Centre, Uppsala University, Science for Life Laboratory, 752 36 Uppsala, Sweden; 3 Department of Behavioural Ecology and Evolutionary Genetics, Max Planck Institute for Ornithology, Eberhard-Gwinner-Straße, 82319 Seewiesen, Germany; 4 School of Biological Sciences, University of East Anglia, Norwich Research Park, Norwich, NR4 7TU UK; 5 Department of Zoology, Faculty of Science, Charles University, Viničná 7, 128 44 Prague, Czech Republic

**Keywords:** Germline-restricted chromosome, Germline/soma genome difference, B chromosome, Non-Mendelian inheritance, Chromosome elimination

## Abstract

Germline-restricted chromosomes (GRCs) are accessory chromosomes that occur only in germ cells. They are eliminated from somatic cells through programmed DNA elimination during embryo development. GRCs have been observed in several unrelated animal taxa and show peculiar modes of non-Mendelian inheritance and within-individual elimination. Recent cytogenetic and phylogenomic evidence suggests that a GRC is present across the species-rich songbirds, but absent in non-passerine birds, implying that over half of all 10,500 bird species have extensive germline/soma genome differences. Here, we review recent insights gained from genomic, transcriptomic, and cytogenetic approaches with regard to the genetic content, phylogenetic distribution, and inheritance of the songbird GRC. While many questions remain unsolved in terms of GRC inheritance, elimination, and function, we discuss plausible scenarios and future directions for understanding this widespread form of programmed DNA elimination.

**Supplementary Information:**

The online version contains supplementary material available at 10.1007/s10577-022-09688-3.

## Introduction

Not all DNA obeys Mendel’s rules of inheritance. One of the best-known cases of non-Mendelian inheritance includes cytoplasmic DNA inheritance. In most animals, the mitochondrial DNA is inherited only through females, although there are exceptions where mitochondrial DNA shows paternal or doubly uniparental inheritance (Sutherland et al. 1998; Zouros 2013; Dégletagne et al. 2021). Other more particular cases comprise the non-random segregation of B-chromosomes, sex chromosomes, centromeres, and various chromosome rearrangements via meiotic drive, which have been observed in many eukaryotes (De Villena and Sapienza 2001a, b; Camacho 2005; Rutkowska and Badyaev 2008; Yoshida and Kitano 2012; Houben 2017; Johnson Pokorná and Reifová 2021). In some organisms, sex chromosomes, sets of chromosomes, or even one of the parental genomes is eliminated during early embryogenesis, deviating from standard Mendelian inheritance (Sánchez 2014; Dedukh and Krasikova 2022). Another increasingly recognized and studied type of non-Mendelian inheritance is seen in the so-called germline-restricted chromosomes (GRCs), present in diverse multicellular organisms such as lampreys and hagfishes (reviewed by Smith et al. 2021), and some dipteran insects (reviewed by Hodson and Ross 2021). GRCs are eliminated from somatic cells through programmed DNA elimination but maintained in the germline and can show different behavior between male and female meiosis. They also sometimes show mitotic instability which results in a variable number of GRC copies in the germ cells. In this review, we critically reflect on the meiotic and mitotic behavior of the GRC of songbirds in light of all the gathered evidence since its discovery, including recent insights into its genetic content and evolution.

The first songbird GRC was described by Maria Ines Pigozzi and Alberto Solari in the zebra finch, *Taeniopygia guttata* (Pigozzi and Solari 1998). It was serendipitously discovered during the comparative study of the meiotic behavior of (sex) chromosomes in female and male birds. Unexpectedly, zebra finch female and male germ cells showed a large additional chromosome (in fact, the largest in the germline karyotype) that was absent in their bone marrow cells (Pigozzi and Solari 1998). This strange chromosome had not been found in other non-passerine bird species examined, i.e., pigeon, domestic chicken, and Japanese quail (Pigozzi and Solari 1999a, b; Pigozzi 2001; Calderón and Pigozzi 2006). The GRC was usually present as two copies in oocytes and as a single copy in spermatocytes (Pigozzi and Solari 2005). Another surprising feature of this GRC was its consistent elimination from the male germ cells soon before the end of meiosis, suggesting exclusive maternal transmission to the progeny (Pigozzi and Solari 1998, 2005), and thus non-Mendelian inheritance.

In its first description, the zebra finch GRC was compared to B chromosomes as they have some characteristics in common. In contrast to the essential A chromosomes, which comprise autosomes and sex chromosomes, B chromosomes are supernumerary and dispensable (Randolph 1928). B chromosomes are known to be present in only a subset of individuals within a species (or even only a subset of cells within an individual) and often accumulated via non-Mendelian mechanisms (reviewed in Johnson Pokorná and Reifová 2021). The GRC resembles a mitotically unstable B chromosome as it is present in only a subset of cells within an individual and because individuals can differ in the number of GRC they carry (Pigozzi and Solari 1998, 2005). However, it distinguishes itself by its consistent presence in the germline and obligatory absence in the soma. In fact, the GRC has been found in germ cells (except for spermatozoa) of all the individuals examined, which strongly suggests that it is indispensable for the germline and is not a standard B chromosome (Camacho 2005). Since its discovery (Pigozzi and Solari 1998), the apparent ubiquity of the songbird GRC, together with its tissue specificity, has been strongly suggestive that the GRC might be important for oogenesis and/or the early stages of spermatogenesis, but dispensable or detrimental for somatic cells. However, due to limitations in sequencing technology, the genetic content and potential function of the songbird GRC remained elusive for decades, until very recently.

## Genetic content of the zebra finch GRC

The zebra finch is currently the only bird species with a sequenced germline genome (Kinsella et al. 2019). Prior to high-throughput genome/transcriptome analyses, the first and only evidence of the zebra finch GRC genomic content was the 27L4 marker identified by Itoh et al. (2009) when comparing DNA from testis and blood of the same individual using random amplified polymorphic DNA–polymerase chain reaction (RAPD-PCR). They showed that this GRC-linked sequence has homology with the short arm of the third-largest chromosome of zebra finch (i.e., chromosome 1 in the somatic reference genome; Kinsella et al. 2019), indicating for the first time that the GRC contains duplicated sequences from the regular A chromosomes. The first coding region of the GRC was only revealed 9 years later and confirmed that the GRC contains paralogous sequences duplicated from the A chromosomes; i.e., by using a subtractive transcriptomic approach, Biederman et al. (2018) characterized a GRC-linked paralog of the *napa* gene (*napa*_*GRC*_) which showed a sequence similarity of 81% to the A-chromosomal paralog (*napa*_*A*_).

Shortly thereafter, Kinsella et al. (2019) compared soma and testis genome sequencing data from three zebra finch individuals with different mitotypes, increasing the catalog to 115 high-confidence GRC-linked genes, three of which (*gbe1*, *robo1*, and *dph6*) were also independently detected by zebra finch GRC microdissection and sequencing (Torgasheva et al. 2019). Strikingly, each of these GRC-linked genes had a paralog on the A chromosome. GRC-linked paralogs were copies of genes located on 19 different A chromosomes and showed different levels of divergence, copy number, and completeness. In contrast to protein-coding genes, the overall abundance of transposable elements and satellite DNA was lower on the GRC when compared to the A chromosomes (Kinsella et al. 2019). Both subtractive transcriptomics (Biederman et al. 2018) and analyses of coverage and single-nucleotide variants (SNVs) comparing testis and soma genome data (Kinsella et al. 2019; Pei et al. 2022) found solely GRC sequences that have an A-chromosomal paralog. These findings indicate that many, if not all GRC sequences, originated from the A chromosomes, without loss of those regions from the A chromosomes. Note that the current catalog of GRC genes is limited to high-confidence genes supported by two independent lines of evidence, SNVs between GRC and A-chromosomal paralogs, and testis/soma coverage differences. Certain GRC-linked genes are likely missing from this catalog, particularly those with low sequence divergence from their A-paralog and those found in a single or low number of copies on the GRC. The latter is mainly due to the presence of somatic cells in the testes, which causes a reduction of the GRC proportion to around one GRC per every three haploid A genomes as inferred from counting testis cells stained by fluorescence in situ hybridization (FISH; Kinsella et al. 2019). Using GRC-linked SNV evidence in a linked-read testis assembly and manual curation, 36 scaffolds with a total length of 1.24 Mb were assigned to the zebra finch GRC, which represents less than 1% of its expected size (Kinsella et al. 2019). Recently, Asalone et al. (2021) developed a bioinformatic approach to reanalyze the testis assembly from Kinsella et al. (2019) and combined soma and testis re-sequencing data from four individuals from Biederman et al. (2018) and Kinsella et al. (2019). They newly identified two protein-coding genes and 733 high-confidence GRC scaffolds in the linked-read testis assembly.

GRC transcription is supported by cytogenetic and transcriptomic analyses. In females, the GRC shows dispersed chromatin as found on the A chromosomes (Pigozzi and Solari 1998) and also forms the typical transcriptionally active loops associated with the phosphorylated form of the RNA pol II with transcriptionally-active chromatin during the meiotic lampbrush chromosomes stage (Torgasheva et al. 2019). However, in males, the GRC appears to be silenced in the primary spermatocytes as suggested by the labeling of the GRC with antibodies against repressive histone modifications such as H3K9me3, H3K9me2, and MacroH2A during meiotic prophase (Schoenmakers et al. 2010; del Priore and Pigozzi 2014). This evidence is consistent with qPCR data which showed higher expression of *napa*_GRC_ in the ovaries than testes (Biederman et al. 2018). Transcriptional activity was also supported by the evidence of 32 GRC-linked genes being expressed in ovaries and 6 in testes (Kinsella et al. 2019). Additionally, the same study confirmed protein expression of 5 GRC-linked genes in adult gonads of both males and females. The gene composition of the GRC appears to be non-random, as the chromosome is enriched in gene ontologies related to embryonic and germline development (Kinsella et al. 2019). Moreover, the GRC was found to be enriched for genes that show high levels of expression in the gonads of chicken (which lack a GRC), suggesting an additional function in the ovaries and testes (Kinsella et al. 2019). These results are based on a subset of high-confidence genes and, therefore, should be interpreted with caution until there is a more complete picture of the GRC content.

The presence and expression of GRC-linked developmental genes indicate that these genes might be functional and should thus exhibit signatures of selection (Kinsella et al. 2019). Biederman et al. (2018) reported the first evidence for purifying selection, i.e., the removal of deleterious amino acid-changing SNVs, acting on a GRC-linked paralogous gene, *napa*_GRC_, by analyzing the ratio between non-synonymous and synonymous SNVs between *napa*_GRC_ and *napa*_A_ of zebra finch and *napa*_A_ of other birds. Kinsella et al. (2019) extended this approach to all GRC-linked genes they identified with ≥ 50 SNVs between GRC and A-chromosomal paralogs (16 genes including *napa*) and identified 9 genes with signatures of long-term purifying selection on the GRC paralog (*bicc1*, *cpeb1*, *efnb1*, *napa*, *pim1*, *pim3*, *rfc1*, *scrib*, *trim71*). Interestingly, one of the GRC paralogs (*puf60*) was found to be under positive selection, i.e., selection to fix beneficial amino acid-changing SNVs. This gene was recently shown to exist in two haplotypes on guppy fish autosomes with opposing effects on male vs. female survival (Lin et al. 2021), suggesting a similar mechanism might have driven positive selection of the zebra finch *puf60*.

Judging from phylogenetic analyses, the GRC-linked paralogs appear to have arrived on the GRC at different times across the evolution of Passeriformes. For instance, *bicc1*_GRC_ suggests an emergence in the oscine songbird ancestor, while *trim71*_GRC_ in the passerine ancestor. In essence, most of the GRC-linked genes emerged very recently, but some of them date back to very early in the songbird diversification, over thirty million years ago (Kinsella et al. 2019). This suggestion was further supported by a comparative cytogenetic analysis of 24 bird species (Torgasheva et al. 2019). Taken together, the GRC appears to be an amalgamation of old and new sequences. A more complete zebra finch GRC assembly and genomic data from more songbirds are needed to conclusively unravel how many genes are ancient and under selection.

## Phylogenetic distribution and interspecies variation

**Fig. 1:**
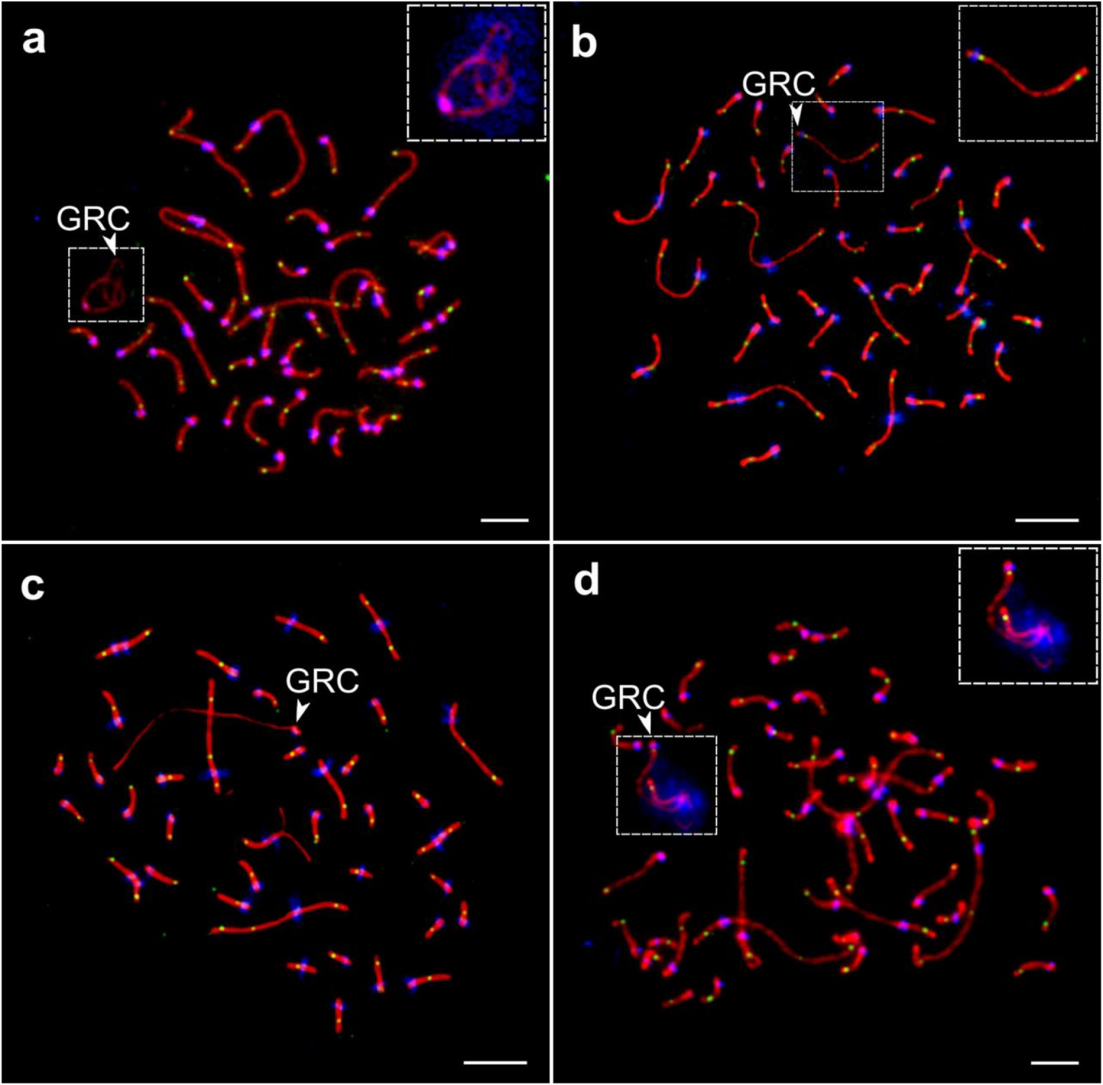
Pachytene spermatocytes of the pale martin (**a**, **d**) and oocytes of the sand martin (**b**, **c**) after immunostaining with antibodies against the synaptonemal complex: SYCP3 (red), centromere proteins (blue), and recombination foci: MLH1 (green). The GRC is usually present as one copy forming a univalent in primary spermatocytes (**a**) and as two copies forming a recombining bivalent in primary oocytes (**b**). Some females contain only one copy forming a univalent in all oocytes (polymorphism) (**c**). Some males contain two partially synapsed GRC copies in few spermatocytes (mosaicism) (**d**). Arrowheads point to GRCs. Inserts show zooms at the GRC with enhanced brightness and contrast. Bar — 5 µm. Individual images taken from Malinovskaya et al. (2020a) via Scientific Reports, License CC-BY-SA 4.0

Until recently, the only other songbird species with a reported GRC was the Bengalese finch, *Lonchura striata domestica* (del Priore and Pigozzi 2014). This changed when Torgasheva et al. (2019) examined pachytene chromosomes immunostained by antibodies against the synaptonemal complex (SC) of 14 songbird species and found a GRC in every one of them. They focused on the male germline because in spermatocytes, the GRC is usually present in one copy as a heterochromatic univalent heavily labeled with antibodies against centromere proteins and therefore is easily distinguishable from the bivalents formed by the regular A-chromosomes (Fig. 1a). In females, the GRC is usually present in two copies forming a recombining bivalent (Fig. 1b). Torgasheva et al. (2019) also showed the absence of the GRC in another 8 non-passerine species from seven different lineages by re-analyzing previously published images of spermatocytes spreads. Recently, the GRC was found in 11 additional songbird species (Slobodchikova et al. 2022; Poignet et al. 2021; Sotelo-Muñoz et al. 2022).

**Fig. 2:**
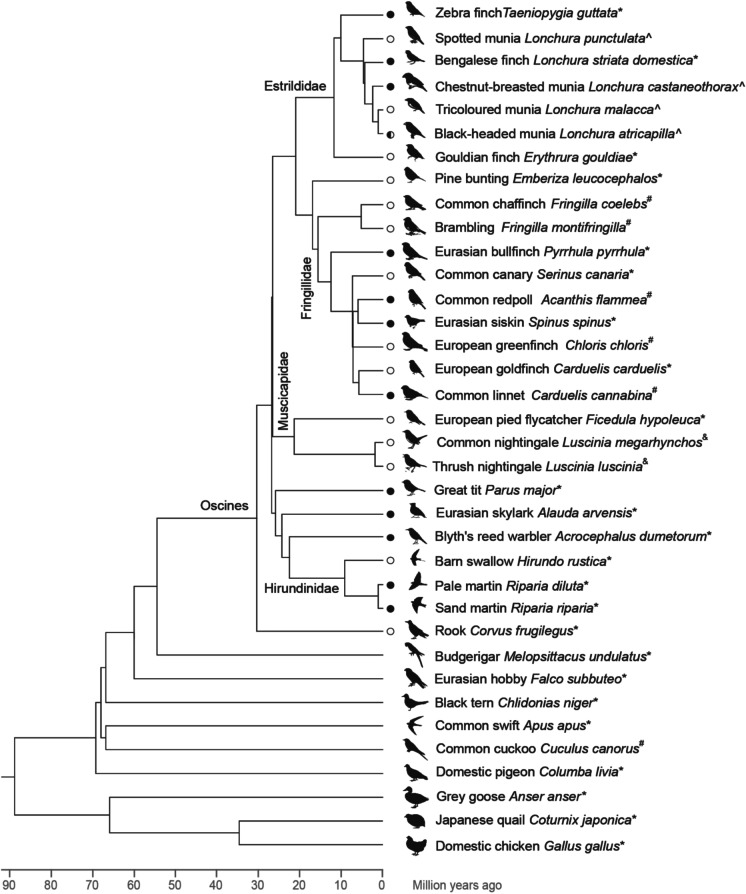
Bird species with cytogenetic evidence for presence or absence of the GRC. Filled circles next to terminal branches indicate species with a macro-GRC and open circles species with a micro-GRC. *data from (Torgasheva et al. 2019), ^#^data from Slobodchikova et al. (2022), ^data from Sotelo-Muñoz et al. (2022), ^&^data from Poignet et al. (2021). The phylogenetic tree is a dated supertree combining Jarvis et al. (2014) for deep avian branches, Oliveros et al. (2019) for deep songbird (Oscines) branches, and TimeTree.org consensus estimates for the remaining more recent branches (Kumar et al. 2017). Note that divergence estimates for deep avian/oscine relationships vary significantly between studies (reviewed by Suh 2016), e.g., the divergence of Estrildidae and Corvidae (common ancestor of the sampled Oscines here) was estimated as 30.6 mya by Oliveros et al. (2019) and 52.9 mya by Ericson et al. (2014). The present supertree shows lower ends of such estimates as a conservative minimum age for GRC emergence, and we proportionally scaled the TimeTree.org branch length estimates within Fringillidae, Muscicapidae, Hirundinidae, and Galliformes relative to each respective outgroup

The presence of a GRC in every songbird species cytogenetically analyzed so far (Fig. 2) suggests a monophyletic origin of the GRC at least in the common ancestor of the sampled songbirds (i.e., spanning the vast majority of Oscines). This hypothesis is supported by the phylogenetic analysis of *bicc1*_GRC_ from the zebra finch relative to *bicc1*_A_ across birds (Kinsella et al. 2019). Although deep-branching Oscines, Suboscines, and Acanthisitti species have not been examined yet, they probably contain a GRC as well, because the zebra finch *trim71*_GRC_ branches outside of *trim71*_A_ from all passerines, suggesting emergence of the GRC paralog in the common ancestor of passerines (Kinsella et al. 2019). Taken together, this indicates that the GRC is present in at least all songbirds, the largest group of birds comprising half of all bird species, and at most in all passerines, nearly two thirds of all bird species (Oliveros et al. 2019).

Cytogenetic analysis revealed that the GRCs show remarkable interspecies variation in size. Torgasheva et al. (2019) classified them broadly as macro- and micro-GRCs (i.e., either belonging to the macro- or microchromosome set of the species). Macro-GRCs were found in 12 species, micro-GRCs in 14 species, and one species showed a mosaic individual containing both macro- and micro-GRCs (Slobodchikova et al. 2022; Poignet et al. 2021; Sotelo-Muñoz et al. 2022). Furthermore, there is no phylogenetic clustering according to GRC size (Fig. 2), suggesting multiple events of massive additions, deletions, and amplifications occurring on the GRC, sometimes even among very closely related species or even within species, such as in the black-headed munia (Sotelo-Muñoz et al. 2022).

In order to estimate divergence of genetic content between GRCs of different species, Torgasheva et al. (2019) prepared whole-GRC microdissected DNA probes. The results of reciprocal FISH with these probes demonstrated substantial genetic divergence between GRCs of different species. The intensity and coverage of the hybridization signal on the GRC decreased when the phylogenetic distance between the species from which the probe was derived and the target species increased (Torgasheva et al. 2019, 2021). Whole-GRC probes derived from different species hybridized with different regions of the A chromosomes. For example, the zebra finch GRC probe labeled the region on the short arm of the third-largest chromosome (i.e., homologous to zebra finch chromosome 1) in all species examined (Torgasheva et al. 2019), while the great tit GRC probe labeled a part of the W chromosome (Torgasheva et al. 2021). These findings suggest that GRCs of different species contain different multiply repeated regions homologous to regions on the A chromosomes.

## The mystery of GRC inheritance

Despite all the information gathered in the almost 25 years since its discovery, it is still not known how the GRC is passed down through the generations. One reason for this is that even though there is a lot of information about the behavior of the GRC in spermatogenesis and in the early stages of female meiosis, knowledge about its behavior during early embryo development and late stages of female gametogenesis is still lacking. In an attempt to explain the differences in GRC copy number between sexes (as well as the occasional variation in GRC copy number in the same sex or even within a single individual), possible scenarios of GRC inheritance have been proposed that will be discussed below.

### Maternal inheritance of a single GRC copy

**Fig. 3:**
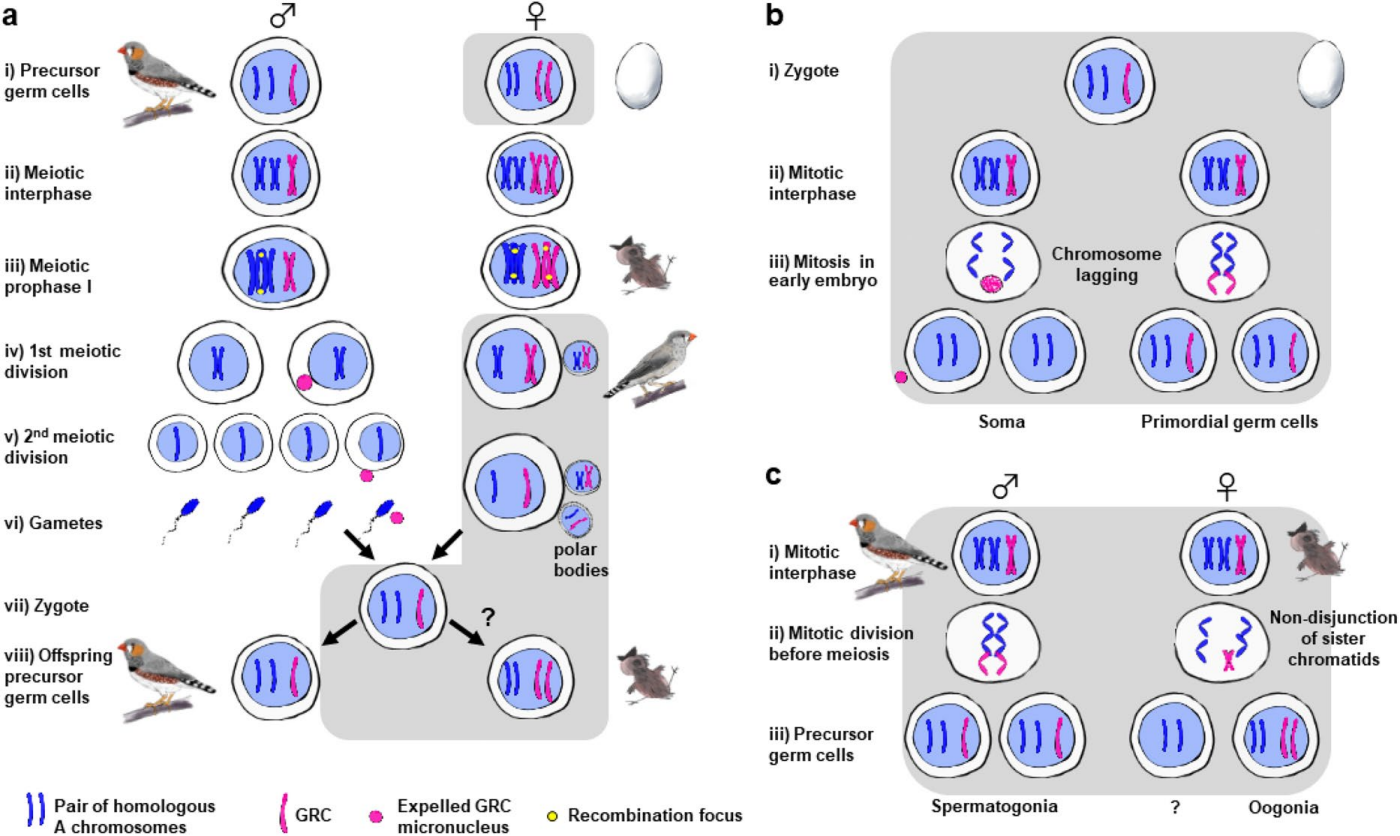
Observed (**a**) and hypothetical scenarios (**b**, **c**) of the meiotic and mitotic behaviors of the GRC. Gray shading indicates the hypothetical scenarios without cytogenetic support. **a** In males, the GRC is typically observed as a single copy in spermatogonia which is eliminated later during spermatogenesis. In female nestlings, the GRC is normally observed as two copies in the primary oocytes that go through regular meiotic recombination. **b** According to Pigozzi and Solari (2005), both male and female zygotes maternally inherit a single copy of GRC. During early mitotic divisions, elimination of the GRC in some cells might result in somatic cells whereas cells with regular mitosis would result in primordial germ cells. **c** After germline/soma differentiation, the single-copy GRC in male primordial germ cell follows regular mitotic behavior. In young females, the GRC might show nondisjunction of sister chromatids during a pre-meiotic cell division, generating one daughter cell containing no GRC and a primary oocyte with two copies of the GRC. Some females or cells may present normal mitosis (i.e., without GRC non-disjunction) resulting in females or cells containing a single-copy of the GRC (polymorphism or mosaicism in females). Also, note that “duplication” of the GRC may occasionally occur during male mitotic division, and this may explain the observation of males with 2–3 copies of the GRC in some of their germ cells (mosaicism in males)

Cytogenetic studies of the GRC have predominantly focused on examining the GRC during meiosis (Fig. 3a). In males, the GRC is normally observed as a single univalent chromosome in spermatogonia (Pigozzi and Solari 1998; del Priore and Pigozzi 2014) and primary spermatocytes (Pigozzi and Solari 2005; Torgasheva et al. 2019). This single GRC is eliminated from the nucleus during the first meiotic division and forms a micronucleus that is later ejected from the cell (Pigozzi and Solari 1998, 2005; del Priori and Pigozzi 2014). In females, the GRC is usually found as two copies (i.e., a paired bivalent, resembling autosomes) that engage in regular crossing-over (Fig. 1b; Pigozzi and Solari 2005; del Priori and Pigozzi 2014). These observations led to the view that the GRC is only inherited from the maternal side (Fig. 3a). A case of maternal inheritance of the GRC was recently reported in F1 hybrids between two different munia species (*Lonchura* spp.) with distinct GRC size, where the F1 hybrids’ GRC matched the size of the maternal species’ GRC (Sotelo-Muñoz et al. 2022). In addition, sequencing of germline samples (testis and ejaculates) from multiple male zebra finches from the same family showed that all brothers shared the same GRC haplotype as the brother of their mother (Pei et al. 2022). However, the apparent uniparental inheritance of a single GRC copy raises the question about when and how a second copy arises in the female, but not in the male.

Pigozzi and Solari (2005) suggested that the sex difference in GRC copy number arises during the germline/soma differentiation. They proposed that in females, nondisjunction of the GRC sister chromatids during a mitotic division may produce a germline progenitor cell with two GRC copies and a somatic progenitor cell with no GRC. In males, one GRC chromatid may be transmitted to the germline progenitor cell and the other one lost (presumably by chromatid lagging during mitotic anaphase), leading to a somatic progenitor cell without a GRC, and a germline with a single GRC (Fig. S1). Pigozzi and Solari (2005) hypothesized that sex-biased expression of cohesin-related genes might be involved in these differences in chromosome behavior. This explanation provides a mechanism to explain not only sex differences in GRC copy number but also how the GRC is lost from somatic cells.

However, it is also possible that the sex-specific difference in GRC copy number does not arise during the differentiation between germline and soma (Fig. 3b), but at a later stage, for example, during mitotic divisions of primordial germ cells (PGCs; Fig. 3c). Nondisjunction of GRC sister chromatids during the mitosis of female PGCs might be a simple mechanism that could lead to one cell without the GRC (which might undergo apoptosis or become a somatic cell) and another cell with two GRC copies that are able to synapse and recombine during meiosis. Such an asymmetrical cell division with GRC non-disjunction would need to be strictly regulated to occur only once and only in females.

### Occasional paternal inheritance of the GRC

While maternal inheritance appears to be the norm, very recent studies suggest that the GRC can occasionally be paternally inherited. Pei et al. (2022) demonstrated that the GRC can occasionally be paternally inherited based on three main findings: (1) a hybrid individual between the two zebra finch subspecies exhibiting mitochondrial DNA from the maternal subspecies but a GRC from the paternal subspecies, (2) a striking topological incongruence between mtDNA and GRC haplotype trees, suggesting that at least some GRC haplotypes were able to cross matriline boundaries, and (3) the presence of the GRC in a small portion of sperm heads. Interestingly, Pei et al. (2022) found that males varied substantially in the proportion of spermatozoa (1–19%) that contained the GRC, and that this pattern is family-specific, with males from the same family showing a consistently low or high proportion of GRC retention in their spermatozoa. This suggests a heritable component for GRC presence in sperm cells. The GRC was also observed in spermatozoa of great tits, although it was rare (in 3 out of 880 spermatozoa; Torgasheva et al. 2021).

It is currently unclear what happens when the zygote receives two copies of the GRC, one of maternal and one of paternal origin. Pei et al. (2022) did not observe any GRC-heterozygous individuals in their sample, but this may simply be a consequence of biparental inheritance being uncommon. If receiving two copies of the GRC would cause substantial problems during embryonic development, such paternal inheritance should be selected against. However, if no problems arise, the strategy of biparental inheritance should rapidly outcompete the strategy of restriction to maternal inheritance, simply because GRCs that are biparentally inherited are more likely to be passed on to the next generation. The observed polymorphism and high repeatability in the effectiveness of GRC elimination during spermatogenesis open up the possibility to study and evaluate the success of the paternal inheritance strategy in the future.

### Polymorphism and mosaicism in GRC copy number and size

The GRC is usually present as two copies in oocytes and as a single copy in spermatocytes (Pigozzi and Solari 2005). However, polymorphism (i.e., variation among individuals) and mosaicism (i.e., variation within the same individual) in GRC copy number have been observed in males and females of some species (Table 1). In the zebra finch (Pigozzi and Solari 1998, 2005) and the sand martin *Riparia riparia* (Fig. 1c; Malinovskaya et al. 2020a), female individuals with a single GRC copy in all their primary oocytes have been found, although in a relatively low proportion (12% of zebra finch females and 17% of sand martin females; Table 1). In the great tit *Parus major*, four of seven females showed mosaicism for GRC copy number, with the majority of primary oocytes containing two GRC copies and the minority (from 2 to 26%) a single copy (Torgasheva et al. 2021). These observations might be explained by the failure of GRC duplication in some or all PGCs during female embryonic development (Fig. 3c). It is plausible that GRC duplication might not be absolutely essential, assuming that in females with a single GRC copy, the unpaired GRC univalent might remain in the egg cell while the polar body does not receive any GRC. The non-negligible frequency in which this has been observed in zebra finch and sand martin populations might mean that females with a single GRC copy do not have a dramatically reduced fitness (compared to females with two GRC copies) and that the duplication of the GRC in females is not under strong selection pressure.

**Table 1:** GRC polymorphism and mosaicism in songbirds. GRC polymorphism refers to between-individual variation in GRC number of the same sex. GRC mosaicism indicates within-individual variation in GRC number

Name	Females				Males			Reference†
	One GRC	Two GRCs	Mosaic*	Total no	One GRC	Mosaic*	Total no	
Zebra finch *Taeniopygia guttata* (ssp. *castanotis*)	1	7	0	8	17	0	17	1, 2, 3, 4, 5, 6
Pale martin *Riparia diluta*	0	3	0	3	2	7** (2–61%)	9	5, 6
Sand martin *Riparia riparia*	4	20	0	24				5, 6
Great tit *Parus major*	0	3	4 (2%-26%)	7	6	1** (4%)	7	5, 7
Bengalese finch *Lonchura striata domestica*	0	3	0	3	8	0	8	5, 8, 9,10
Barn swallow *Hirundo rustica*	0	3	0	3	5	0	5	5,11
Pied flycatcher *Ficedula hypoleuca*	0	2	0	2	1	1 (93%)	2	5,9
Blyth’s reed warbler *Acrocephalus dumetorum*					1	0	1	5,9
Gouldian finch *Chloebia gouldiae*					1	0	1	5,9
Eurasian siskin *Spinus spinus*					2	0	2	5,9
European goldfinch *Carduelis carduelis*					1	0	1	5,9
Eurasian skylark *Alauda arvensis*					1	0	1	5,9
Pine bunting *Emberiza leucocephalos*					1	0	1	5,9
Eurasian bullfinch *Pyrrhula pyrrhula*					2	0	2	5,9
Common canary *Serinus canaria* (f. *domestica*)					1	0	1	5,9
Rook *Corvus frugilegus*					1	0	1	5,9
Common linnet *Carduelis cannabina*					1	0	1	9
Common redpoll *Acanthis flammea*					1	0	1	9
European greenfinch *Chloris chloris*					1	0	1	9
Brambling *Fringilla montifringilla*					1	0	1	9
Common chaffinch *Fringilla coelebs*					1	0	1	9
Black-headed munia *Lonchura atricapilla*					1	1*** (5%)	2	10
Chestnut-breasted munia *Lonchura castaneothorax*					2	0	2	10
Spotted munia *Lonchura punctulata*					2	0	2	10
Tricoloured munia *Lonchura malacca*					2	0	2	10
Thrush nightingale *Luscinia luscinia*					2	0	2	12
Common nightingale *Luscinia megarhynchos*					2	0	2	12
Total	5	41	4	50	67	9	76	
Frequency	0.10	0.82	0.08		0.88	0.12		

^*^Number of mosaic birds and the percentage of cells that differed from the norm for that sex (i.e., 2 for female and 1 for male). **The frequencies of cells carrying more than 1 GRC were estimated from both premeiotic and meiotic cells. One of the *Riparia diluta* male also had 3 GRCs in 6% of pachytene cells. ***In addition to 5% differing in GRC number, cells also differed in GRC size with 37% micro-GRC and 58% macro-GRC. †References: 1: Pigozzi and Solari (1998); 2: Pigozzi and Solari (2005); 3: Schoenmakers et al. (2010); 4: Goday and Pigozzi (2010); 5: Torgasheva et al. (2019); 6: Malinovskaya et al. (2020a); 7: Torgasheva et al. (2021); 8: Priore and Pigozzi (2014); 9: Slobodchikova et al. (2022); 10: Sotelo-Muñoz et al. (2022); 11: Malinovskaya et al. (2020b); 12: Poignet et al. (2021). Note that the absolute sample size of Schoenmakers et al. (2010) is unknown, at least one male and one female was used

In males, mosaicism in GRC copy number has been observed in four species (Table 1). In pale martins, *Riparia diluta*, seven out of nine analyzed males showed GRC copy number mosaicism in primary spermatocytes (Fig. 1d; Malinovskaya et al. 2020a). In these males, most primary spermatocytes had a single GRC copy, but spermatocytes with two or even three copies were also observed. Similar observations were described in the great tit (Torgasheva et al. 2021) and black-headed munia (Sotelo-Muñoz et al. 2022), wherein some spermatocytes with two GRC copies were observed. In one of the two analyzed individuals of European pied flycatcher, most of the primary spermatocytes surprisingly carried two GRCs (Slobodchikova et al. 2022). The relatively high number of species in which such mosaicism has been observed could suggest that the segregation of the GRC during mitosis is often unstable in males. Occasional non-disjunction of GRC sister chromatids during mitotic divisions of PGCs might be the reason why some spermatocytes carry more than a single GRC copy, as it has been observed for B chromosomes in some species (Nur 1963; Jones 2018). It is plausible that such variability may be inconsequential since all GRC copies seem to be canonically eliminated during spermatogenesis (Pigozzi and Solari 2005; del Priori and Pigozzi 2014; Sotelo-Muñoz et al. 2022).

Polymorphism and mosaicism were observed not only for GRC copy number but also for GRC size. In one male black-headed munia, a small proportion of spermatocytes contained two GRCs, either a micro-GRC and a macro-GRC or else two micro-GRCs (Sotelo-Muñoz et al. 2022). This suggests that significant variation in the GRC size may exist not only between species but also within species and even within a single individual. Sotelo-Muñoz et al. (2022) suggested several mechanisms which could explain the origin of within-species polymorphism in GRC size. For example, fragmentation of the GRC during its elimination from the spermatocytes followed by paternal inheritance of the GRC fragment can lead to the origin of a smaller GRC in a population. A shorter GRC might also be the result of GRC fragmentation and loss of its parts during germline mitotic divisions. This sort of mutation would normally not be tolerated by the cell in standard A chromosomes, but given the enormous variability in GRC size even among closely related species, it is possible that large parts of this chromosome are in fact non-essential and thus their loss might not have large effects on their carrier’s fitness. At the same time, additions of new sequences to the GRC from standard chromosomes may be well tolerated as the presence of the GRC only in the germline reduces the pleiotropic effects of such mutations. It is plausible that once polymorphism in the GRC size exists in the population, occasional inheritance of two GRCs of different sizes by a single zygote and unstable mitotic inheritance of these GRCs may result in the observed GRC size mosaicism.

Currently, the frequency of polymorphism and mosaicism for GRC copy number and size in songbirds is difficult to estimate. Most species have only had a few individuals analyzed, and most of these individuals were males, making estimates for females especially uncertain. However, the data obtained to date indicates that polymorphism and mosaicism in GRC copy number could be relatively frequent across songbird species (Table 1).

### Female meiotic drive and maternal inheritance of two GRC copies

An alternative explanation for where the two GRCs in females come from, as well as why polymorphism and mosaicism for GRC number occur, was proposed by Malinovskaya et al. (2020a). They suggested that zygotes of both males and females can already contain two GRC copies. Both copies would be inherited from the mother due to nondisjunction of GRC homologs in the first meiotic division (MI) and their preferential segregation into the egg (i.e., meiotic drive). During germline development, germ cells can actively eject or passively lose one of the GRCs. Since male germ cells undergo a much higher number of mitotic divisions before entering meiosis, they would be more likely to lose one of the GRCs and contain a single copy in the pachytene cells. This scenario does not exclude the possibility that zygotes with one GRC copy occasionally arise via normal segregation of two GRCs in female meiosis I. A single GRC could also be inherited from mothers carrying a single GRC in their pachytene cells, which would explain why some females have only one GRC.

This scenario could potentially explain sex differences, polymorphism, and mosaicism in GRC copy number. However, it depends on the validity of its key assumption: meiotic drive via nondisjunction and preferential segregation of both GRC homologs into the secondary oocyte (and then to the egg cell after normal segregation in meiosis II). In birds, the only known asymmetric divisions, which could provide a high efficiency of GRC accumulation, occur during female meiosis. Both polar bodies are formed at the periphery of the oocyte; therefore, if GRC homologs do not separate, they have a high chance to remain in the egg. Indeed, meiotic drive of B chromosomes during asymmetrical MI has been documented in females of many non-avian species (Hewitt 1976; Nur 1977; Nur and Brett 1985; Cano and Santos 1989; Santos et al. 1993). However, these studies mostly described the drive of a single B chromosome, which formed a univalent. Meiotic drive of GRC in MI requires nondisjunction of properly synapsed GRC bivalents. Malinovskaya et al. (2020a) suggested that nondisjunction can be facilitated by the extreme polarization of chiasmata positions in GRC bivalents. In females of all three species carrying macro-GRCs studied to date (zebra finch, sand martin, and great tit), recombination occurs in one or both ends of GRC bivalents (Fig. 1b; Pigozzi and Solari 2005; Malinovskaya et al. 2020a; Torgasheva et al. 2021). Such a polarized distribution of chiasmata is associated with an increased frequency of chromosome nondisjunction at the first meiotic division in other organisms (Sears et al. 1995; Koehler et al. 1996; Hassold and Hunt 2001).

However, recent observations reporting the lack of heterozygosity in zebra finch male siblings, which share the same GRC haplotype as their uncle from their maternal side (Pei et al. 2022), contradict the assumption that two homologous GRCs are transmitted to the progeny. They must have accumulated noticeable differences if passed through many generations and recombined in limited regions. Nevertheless, female meiotic drive at MI and inheritance of two GRCs from females could possibly occur at least in some species or individuals.

**Fig. 4:**
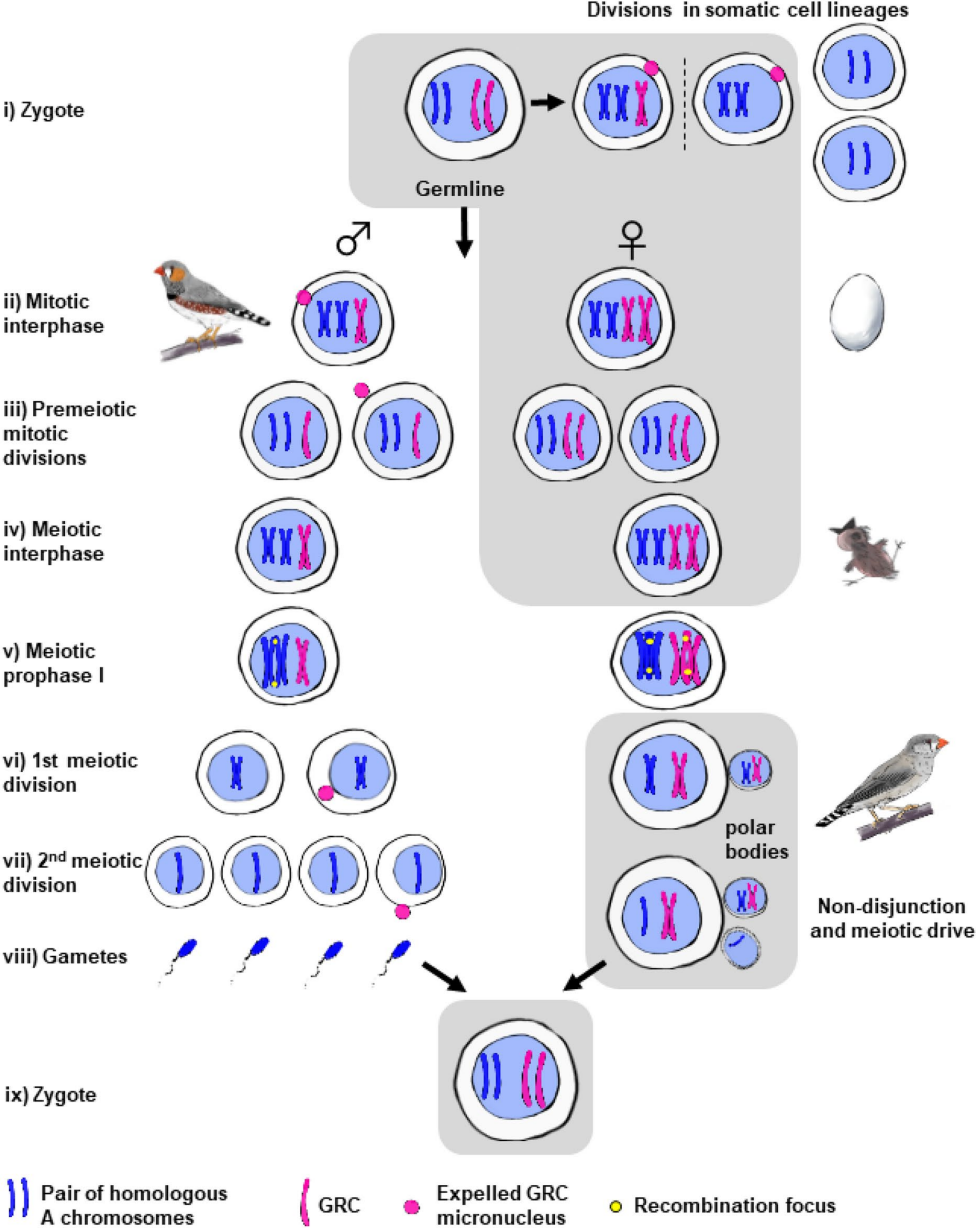
Hypothetical scenario of GRC transmission via meiotic drive in females and programmed elimination in males. Gray shading indicates the hypothetical events without cytogenetic support. According to the scenario, both males and females normally inherit two GRC copies from the mother. Both GRC copies are passively lost or actively eliminated in somatic cell lineages of both sexes. In male germline, one of the GRC copies is eliminated during early pre-meiotic mitotic divisions, the other during meiotic divisions. Delayed elimination of the first GRC copy may lead to mosaicism in males. In female germline, two GRC copies follow regular mitotic behavior. They form a bivalent and recombine in the meiotic prophase, properly segregate in the first meiotic division, non-disjoin and preferentially segregate to the egg cell in the second meiotic division (MII). Some females may present proper segregation in MII resulting in zygotes with single GRC, which can explain polymorphism in females

Zygotes with two GRCs can also occur via non-disjunction of GRC sister chromatids and their preferential segregation to the egg cell in the second meiotic division (MII; Fig. 4). Meiotic drive in MII, although less intuitive than in MI, can also occur due to the asymmetrical geometry of this division (reviewed in Clark and Akera 2021). One may speculate that meiosis-specific cohesins or other meiotic players controlling the correct separation of sister chromatids or centromeres might be involved in the GRC nondisjunction at MII in a similar way as has been described for B chromosome drive during the first pollen mitotic division (Ruban et al. 2020). Occasional normal disjunction in MII and rare nondisjunction during premeiotic mitoses can explain how polymorphism and mosaicism for GRC copy number arise in females. In addition, under this scenario even females with a single GRC would produce gametes with two GRC copies, thereby maintaining the polymorphism in GRC copy number in the population.

### GRC elimination from somatic cells and male germ cells

Another question is how the GRC is eliminated from somatic cells during early embryogenesis and from male germ cells in spermatogenesis. Currently, the mechanisms of GRC elimination from somatic cells remain entirely unknown. Pigozzi and Solari (2005) hypothesized that GRC elimination from somatic cells occurs via different mechanisms in males and females. In females, nondisjunction of the GRC chromatids and their segregation to a germline progenitor cell would leave a somatic progenitor cell without a GRC. In males, lagging of one of the GRC chromatids during mitotic anaphase would lead to a somatic progenitor cell without a GRC and a germline progenitor cell with a single GRC (Fig. S1). Such sex-specific differences in GRC behavior would, however, require sex differences in gene expression already at early stages of embryo development when the germline is determined. Alternatively, the GRC might be epigenetically modified in both sexes and marked for elimination from somatic cells in a similar way as it has been observed in spermatogenesis (del Priore and Pigozzi 2014; Malinovskaya et al. 2020a). Cytological observations of the earliest stages of songbird embryonic development are needed to shed light on the details of the GRC elimination from somatic cells.

A few pilot studies on mechanisms of GRC elimination from male germ cells during spermatogenesis have already been published (del Priore and Pigozzi 2014; Goday and Pigozzi 2010; Malinovskaya et al. 2020a; Schoenmakers et al. 2010). They showed that from the very beginning of meiotic prophase, the single GRC is heterochromatic in primary spermatocytes, marked with specific histone modifications during prophase (e.g., H3K9me3, H3K9me2, and MacroH2A), and shifted to the nuclear periphery. The GRC is then observed in the cytoplasm of secondary spermatocytes, suggesting that its elimination from the nucleus occurs during the first meiotic division. Later, the GRC is seen as a micronucleus in the cytoplasm of secondary spermatocytes and young spermatids, and finally expelled from the cells. A similar mechanism might be involved in GRC elimination from male germ cells during their pre-meiotic mitotic division if a zygote receives two GRC copies (see above). This is supported by cytological observations of pale martin spermatogonia containing two GRC copies, one of which is located within the nucleus and the other one is moved to the cell periphery and almost expelled (Fig. 5c in Malinovskaya et al. 2020a).

## Evolutionary significance

The likely presence of the GRC in > 5,000 songbird species with mostly two copies in females and one copy in males, and the signatures of long-term purifying or positive selection on some zebra finch GRC-linked genes make it very tempting to speculate about the evolutionary significance of GRCs. As our conservative minimum estimate of GRC emergence is 30 mya in the ancestor of songbirds (Fig. 2), we emphasize that such an ancient origin of the songbird GRC may lead to the difficulty of distinguishing between the (potentially different) reasons that the GRC might have originally evolved for vs. what it might be doing now.

Kinsella et al. (2019) defined 115 high-confidence genes out of a total of 267 candidate genes located on the zebra finch GRC, suggesting that the GRC is a gene-rich chromosome with an amalgam of paralogs from across the A chromosomes. While the remaining candidate genes await further verification, the 115 high-confidence genes alone provide ample opportunity to deliberately pick genes that point in different speculative directions:

i. What if the GRC is simply a very successful parasitic B chromosome? B chromosomes are supernumerary dispensable chromosomes, which occur in a single or multiple copies in the cell and often show unstable meiotic and mitotic inheritance, which may result in their loss from the population. Because they usually do not provide any advantage to their carriers (but see Johnson Pokorná and Reifová 2021 for exceptions) and can be even harmful especially if they occur in odd copy numbers (Camacho et al. 2004), many B chromosomes evolved mechanisms to increase probability of their inheritance in a selfish way (Jones 2018). Some B chromosomes show meiotic drive increasing the chance of their transmission to the germ cells, while others, for example, show mitotic drive leading to the preferential segregation of both B chromosome chromatids to the germline (gonotaxis). Similarities between B chromosomes and GRCs have been previously noted (Johnson Pokorná and Reifová 2021) and it seems plausible that the latter might be a way of long-term stabilization of a B chromosome, i.e., a GRC would be a B chromosome with stable germline inheritance and somatic elimination. Such a B-chromosomal origin would be consistent with the female bias in GRC inheritance, akin to meiotic drive of B chromosomes often through the asymmetry of female meiosis (Jones 2018; Clark and Kocher 2019). The songbird GRC may represent such an extraordinarily successful B chromosome, which found a way to prevent its loss, albeit retaining many B chromosome characteristics including unstable mitotic inheritance and possibly female meiotic drive. In line with this, it is worth noting that the zebra finch GRC contains paralogs of *cenpj* (Kinsella et al. 2019), a gene involved in centriole architecture (Hatzopoulos et al. 2013) and thus compatible with speculation about the GRC ensuring its own transmission. In terms of chromosome organization, a recent high-quality assembly of the B chromosome of the Mexican cavefish *Astyanax mexicanus* bears striking resemblance to the GRC as it also contains high-copy paralogs from across the A chromosomes (Imarazene et al. 2021).
ii. What if the GRC is involved in sexual conflict (resolution) or is even a sex chromosome? Stöck et al. (2021) recently noted that the usual songbird situation of one GRC in males and two GRCs in females resembles a germline-limited X0/XX sex chromosome system, i.e., on top of the organism-wide ZZ/ZW sex chromosome system of birds. For the at least six Z-paralogous genes on the GRC (Kinsella et al. 2019), this would effectively balance the dosage of these genes between males and females given the lack of global dosage compensation in birds (reviewed in Stöck et al. 2021). Considering that GRC inheritance might not be strictly maternal leaves additional room for sexual conflict or resolution thereof (Pei et al. 2022), and the presence of the *puf60* gene on the zebra finch GRC with signatures of positive selection (Kinsella et al. 2019) brings to mind recent evidence from guppies where different alleles of this A-chromosomal gene link to male vs. female survival in the wild (Lin et al. 2021).
iii. What if the GRC is involved in germline development or even a germline determinant? Kinsella et al. (2019) noted that several GRC-linked genes, including the so far oldest genes *trim71*_GRC_ and *bicc1*_GRC_, are involved in cell differentiation and germline development. *trim71*_GRC_ and *bicc1*_GRC_ have been under long-term purifying selection, *bicc1*_GRC_ mRNA was found expressed in adult ovary, and the 115 high-confidence GRC-linked genes are enriched for genes involved in female gonad development (Kinsella et al. 2019). While it remains unclear whether the GRC is expressed during early embryo development as GRC expression analyses have been limited to adult male and female gonads, these patterns would be in line with the explanation for programmed DNA elimination invoked for lampreys: The germline limitation of specific genes (or specific paralogs) might allow the evolution of germline-beneficial functions without detrimental effects when misexpressed in the soma (Smith et al. 2012; Smith 2017), i.e., minimizing antagonistic pleiotropy. In line with this hypothesis, GRCs might be an irreversible means of avoiding germline gene misexpression in the soma, with the potential to act as a germline determinant (Kinsella et al. 2019). Germline gene misexpression in species without programmed DNA elimination (e.g., humans) has led to the notion of “cancer/testis antigens” as a form of antagonistic pleiotropy through oncogenesis in the soma (Simpson et al. 2005; Sandhu et al. 2021).

However, what if the GRC is none of these or rather several of these? Until there is a full understanding of GRC gene content across and within species, we recommend that speculation based on cherry-picked genes, no matter how tempting, should be taken with a grain of salt.

## Conclusion and future directions

Although the songbird GRC has been known for nearly 25 years, only high-throughput multi-omics and comparative cytogenetic studies during the last 5 years have elevated GRCs from a niche oddity of zebra finches to a general phenomenon of all songbirds if not all passerines, i.e., at least half and up to two thirds of all 10,500 bird species. While many mysteries remain, especially with regards to when and how the GRC is transmitted or eliminated, we are positive that the newly developed genomics and cytogenetic approaches will allow high-resolution tracing of GRC presence or absence across embryogenesis and gametogenesis of zebra finches over the next years. To understand the evolutionary significance and potential adaptive value of the GRC, characterizing within-species and between-species GRC genetic diversity and gene content is needed. Seeing that GRCs of thousands of passerine species await cytogenetic and genomic characterization, the songbird GRC serves as an important reminder that multitudes of GRCs or other germline/soma genome differences are waiting to be discovered across the tree of life.

## Supplementary Information

Below is the link to the electronic supplementary material.

Supplementary file1 (DOCX 113 KB)

## Abbreviations

GRC: Germline restricted chromosome
H3K9me2: Histone H3 dimethylated at lysine 9
H3K9me3: Histone H3 trimethylated at lysine 9
MI: Meiosis division I
MII: Meiosis division II
MLH1: MutL homolog 1
SNV: Single nucleotide variant
SYCP3: Synaptonemal complex protein 3
