# Supplementary material for: Mendelian nightmares: the germline-restricted chromosome of songbirds
Source: Chromosome Res. 2022 Apr 13;30(2-3):255–72. doi: 10.1007/s10577-022-09688-3 (PMC9508068; doi:10.1007/s10577-022-09688-3)
Supplement: Supplementary file 1 — Supplementary file1 (DOCX 113 KB) [file 10577_2022_9688_MOESM1_ESM.docx]

### **Supplementary Material**


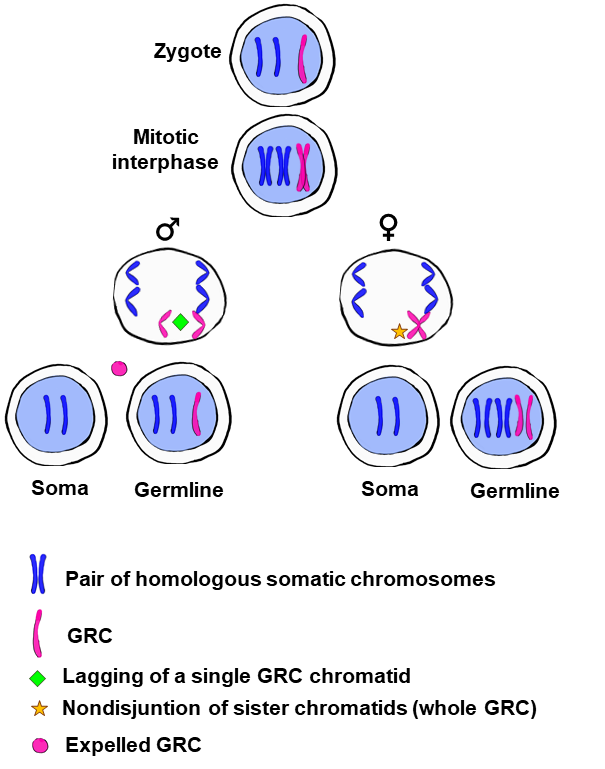


**Figure S1.** The possible sex-dependent behaviour of the GRC during early embryo development, explaining differences in the number of GRC copies between males and females proposed by Pigozzi and Solari (2005). According to Pigozzi and Solari (2005), the zygote receives a single copy of the GRC from the mother, but females and males end up with two or one copy – respectively – due to a sex dependent behaviour during the mitosis previous to the soma/germline differentiation. In females, nondisjunction of sister chromatids (the whole GRC) would generate a germline cell with two GRC copies and a somatic cell without a GRC; in males, the lagging of one of the sister chromatids of the GRC would generate a germline cell with a single GRC copy and a somatic cell with no GRC.
